# DNAseq Workflow in a Diagnostic Context and an Example of a User Friendly Implementation

**DOI:** 10.1155/2015/403497

**Published:** 2015-06-07

**Authors:** Beat Wolf, Pierre Kuonen, Thomas Dandekar, David Atlan

**Affiliations:** ^1^University of Applied Sciences and Arts of Western Switzerland, Perolles 80, 1700 Fribourg, Switzerland; ^2^University of Würzburg, Am Hubland, 97074 Würzburg, Germany; ^3^Phenosystems SA, 137 Rue de Tubize, 1440 Braine le Chateau, Belgium

## Abstract

Over recent years next generation sequencing (NGS) technologies evolved from costly tools used by very few, to a much more accessible and economically viable technology. Through this recently gained popularity, its use-cases expanded from research environments into clinical settings. But the technical know-how and infrastructure required to analyze the data remain an obstacle for a wider adoption of this technology, especially in smaller laboratories. We present GensearchNGS, a commercial DNAseq software suite distributed by Phenosystems SA. The focus of GensearchNGS is the optimal usage of already existing infrastructure, while keeping its use simple. This is achieved through the integration of existing tools in a comprehensive software environment, as well as custom algorithms developed with the restrictions of limited infrastructures in mind. This includes the possibility to connect multiple computers to speed up computing intensive parts of the analysis such as sequence alignments. We present a typical DNAseq workflow for NGS data analysis and the approach GensearchNGS takes to implement it. The presented workflow goes from raw data quality control to the final variant report. This includes features such as gene panels and the integration of online databases, like Ensembl for annotations or Cafe Variome for variant sharing.

## 1. Introduction

Next generation sequencing (NGS) technologies saw a rapid progression over recent years and improved in many aspects since their introduction. The data quality, the length of the sequences, and the speed at which the data is generated improved massively all while decreasing the costs associated with the technology [1]. Cost reductions and quality improvements now allow the technology to be used in a diagnostic setting, replacing older technologies such as Sanger sequencing [2]. An increasing number of laboratories are either considering or already implementing NGS for routine diagnostics procedures. Next generation sequencing has many use-cases in diagnostics and, depending on the analysis, different types of information are searched for in the sequencing data. A very common type of analysis is the search for SNPs (single-nucleotide polymorphisms) and small indels (insertions and deletions) in patient DNA. With the increasing number of documented variations in well-known disease related genes, this type of analysis became very common. Increasingly, other types of analyses are also used in diagnostics, such as the detection of structural variations, which became possible with the increased quality of sequencing data. The sequencing data, which can come from various sequencing technologies, can be either targeted, such as targeted gene panel sequencing and whole exome sequencing (WES), or nontargeted like whole genome sequencing (WGS). Those different approaches to sequence the patient's genome produce a varying amount of data [3] and are suited for different types of analyses. The complexity of the analysis increases with the amount of data to process, both from a computational point of view and from a human resources point of view. The general tendency today is to move away from targeted sequencing towards WGS, as this removes the need for targeted amplicon libraries, as well as making it easier to reanalyse a sample at a later date without the need to resequence it. This tendency to increase the amount of sequenced data creates increasing challenges for smaller diagnostics laboratories. They often do not have the required computing infrastructure or technical know-how to handle this type of data. This is especially true in emerging markets which do not always have the required financial and human resources as well as the extensive know-how required to set up their own analysis pipeline.

Today many types of NGS data analysis exist, all of them answering different sorts of questions about a sample. One example being copy number variation (CNV) analysis suited to replace traditional MLPA (multiplex ligation-dependent probe amplification) analysis. Another one is de novo sequence alignment to detect large changes in the genome, for example, in cancer patients. But ultimately the detection of SNPs and small indels in a sample based on a standard reference sequence remains the most prevalent type of analysis. This is why, in the context of this paper, we limit our study to this type of analysis: the detection of SNPs and small indels in targeted or nontargeted resequencing. Although this type of analysis can be approached in various ways, it can be described as a set of generic steps that remain common to most approaches. The next chapter describes the commonly used steps in the analysis process, providing examples of existing tools that can be used to perform them. 

**Figure 1:**
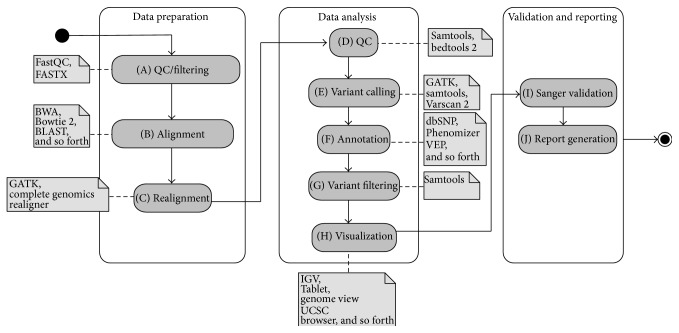
UML activity diagram of a NGS diagnostics workflow separated into different steps. A collection of the available tools for every step is mentioned.

*NGS Data Analysis*. This chapter describes a common NGS data analysis workflow used in diagnostics.  Figure 1 shows an overview of the described workflow, using a Unified Modeling Language (UML) diagram.

*(A) QC/Filtering*. The analysis starts with the quality control of the raw sequencing data to determine if the sequencing process adheres to the defined quality requirements. Different metrics, such as the amount of reads sequenced, the length distribution of the sequenced reads, and their quality, are verified and compared to the expected values of the specific sequencer technology that has been used. If the quality of the sequenced data does not match the required quality, the sample is usually resequenced. To verify the quality of the raw data, one commonly used tool is FastQC [4], which generates a comprehensive list of statistics of the raw data. During this initial quality control stage, the raw sequencing data is often filtered, discarding reads that do not match the defined quality criteria. This raw data filtering is commonly done with the FASTX toolkit [5], which allows removing reads that do not match the required quality, by removing low quality reads or reads that are too short. Simultaneously to filter the raw data based on its quality, it is increasingly common to preprocess the data, for example, to remove barcodes attached to the sequenced reads. Barcodes are added to the sequenced reads when multiple samples are sequenced together and need to be separated again before the analysis. This splitting based on barcodes can also be done using a tool like FASTX. 

*(B) Alignment*. The verified raw sequencing data is then aligned against the human reference genome. During this process every sequenced read is placed at the most fitting position on the reference genome. What the most fitting position will depend on the algorithm and parameters used. This process is specially complicated when aligning sequences from repeated regions or containing insertions and deletions. The alignment process can be done by using one of many available tools [6]. Many of those tools are actively developed and possess their individual qualities, depending on the use-case and sequence data to be aligned. Some of the more commonly used tools for sequence alignment are BWA [7], Bowtie 2 [8], or BLAST [9]. This list is not exhaustive and further information on the subject can be found in [6]. 

*(C) Realignment*. In addition to the alignment, tools exist to improve the quality of an existing alignment, for example, with local indel realignment. Tools like GATK [10] and the Complete Genomics realigner [11] provide this functionality. This and the previous* Alignment* analysis step are some of the heaviest in terms of computing power and require a good computing infrastructure to be performed in a short time frame. This problem is accentuated with the increasing amount of produced raw data and expected qualities of the alignment, notably for indels. 

*(D) QC*. Another round of quality control is usually performed after the final alignment has been created. It is used to verify that the sample has been sequenced correctly, in particular in the regions of interest, like the genes associated with the patients phenotype. Several tools allow the extraction of the information needed for this step, such as samtools [12] or bedtools 2 [13]. They allow the user to get metrics such as the number of aligned reads and the coverage of the regions of interest out of the alignment file. If the quality of the alignment is not sufficient at this stage, it can be decided to resequence the sample. This step of quality control is critical in a diagnostic setting where it is necessary to show how well the regions of interest for a specific sample have been covered. It is especially important for cases where no variants of interest can be found in the specified zones and thus a negative findings report needs to be justified. 

*(E) Variant Calling*. After the raw sequencing data has been aligned and its quality verified, SNPs and indels can be called. The process of variant calling compares the aligned sequences to the reference sequence in order to create a list of variants present in the sample. This list can be filtered on different criteria, such as minimum coverage at the variant position or the frequency of the variant allele in the sample. Commonly used tools are GATK [10], samtools [12], and Varscan 2 [14]. Different variant callers follow different underlying models, resulting in differences in the called variants [15]. Variant calling usually yields a large amount of variants, even when restricting the calling to only output variants with a minimum quality and probability not to be a sequencing artifact. 

*(F) Annotation*. This is why in the next step the list of the variants is annotated with more information that is pertinent to the analysis being done on the sample. This includes the detection of variants present in public databases, such as dbSNP [16], or the information about which genes are affected and their predicted effects on those genes. Various tools exist to annotate variants, either locally or through a web service. Some of the more popular ones are the Variant Effect Predictor (VEP) from Ensembl [17] and Phenomizer [18] that add clinically relevant information to the detected variants. Once the annotated list of variants has been created, the phenotype relevant variant has to be identified. 

*(G) Variant Filtering*. Analysis specific filters allow the user to filter the number of variants down to a smaller number. One example is to only keep variants affecting genes with phenotypes matching the ones of the patient and that have a predicted consequence on the gene function. The exact type of filters to reduce the amount of candidate variants varies from case to case, depending on the available information. The filtering is often done using bcftools, which is part of the samtools project [12] or vcftools [19]. If during this step no clinically relevant variant related to the current analysis can be found, depending on the analysis protocol, the filters may be relaxed. This allows the user to include, for example, variants on genes where the relation with the current phenotype is less certain, or variants with uncertain consequences on a known disease gene. 

*(H) Visualization*. Once a list of candidate variants has been identified, it is common to visually verify them in a genome browser. The genome browser displays the sequenced reads around the variant and allows the user to verify the quality of the data around that position, allowing the user to identify and discard variants originating from sequencing errors. This is done using one of the various genome browsers installed locally such as IGV [20], Tablet [21], or Genome view [22]. There also exist web-based genome browsers, such as the UCSC [23] or Ensembl [24] genome browsers. 

*(I) Sanger Validation*. Before submitting the final findings to the clinician, it is still common practice to validate the identified variants through Sanger sequencing. While future advances in NGS technologies might make this step redundant, it is still considered the gold standard for variant validation. 

*(J) Report Generation*. After visually inspecting and validating the variants, the identified variants or the lack thereof are documented in a final report that is sent off to the clinician to allow him to take decisions on the case.

The described process can be done by taking several approaches. A common approach is to use a selection of the previously described tools to perform the individual analysis steps. The different tools are usually connected together through a series of automated scripts, performing the required analysis. The creation of the resulting pipeline is a complex technical task, as it requires insight in the working of the different tools and the ability to chain them together. This is a nontrivial task, as not all tools follow the same standards in terms of data file formats and handling. Nonetheless, this is a very common approach which resulted in a variety of custom made NGS analysis pipelines, either private or public [25].

Complete software packages like CLCBio [26], NextGENe [27], or the web-based Galaxy [28] also exist. They allow the user to combine many of the mentioned analysis tools, without having to worry too much on how to connect the different tools to perform the analysis. Those tools are focused on giving the user the possibility to automate the analysis process as much as possible, abstracting the complexity of the analysis. GensearchNGS belongs to this family of tools, with its own approach and focus on the issues of smaller laboratories. It focuses less on the possibility to generate a custom automated pipeline but more on an intuitive interface to handle the data-files to perform the analysis in a predefined pipeline optimised for diagnostics.

The features of GensearchNGS are limited to those needed to perform the presented NGS diagnostics workflow. This means the overall feature set is smaller than that of other complete software packages, especially CLCBio or Galaxy, which both offer a wide variety of functionalities. Having a limited feature set allows for a cleaner and more focused user interface, one of the major goals of GensearchNGS, something that is highly appreciated by current users of GensearchNGS.

As will be discussed in the following chapters, GensearchNGS uses either external applications through plugins or custom implementations of standard algorithms for the different analysis steps. The resulting analysis quality is therefore comparable to the one observed when using those tools separately by hand or through a software package like Galaxy. 

*Approach*. The issues faced by small laboratories when using the previously mentioned tools, which are often limited in terms of computing and human resources, are various. Based on our experience, the biggest issue faced by smaller laboratories is the lack of technical know-how and dedicated bioinformaticians. The usage and configuration of many of the described data analysis tools require a dedicated employee which not available in every laboratory. This is especially true in emerging markets where many monetary restrictions are in place. The approach of solutions like Galaxy and CLCBio to this problem is to abstract the usage of the underlying analysis tools and provide a way for the user to automate the analysis process. While the abstraction of the underlying tools reduces the technical complexity of the analysis process, this is often not enough to make the analysis process more accessible. The cited tools can be used for many other types of analyses than the ones looked at in this paper, which leads to a much more complex user interface and often overwhelms the user with many options. The cited tools also focus more on the ability to create automated analysis pipelines, leading to very little interactivity between the user and the data.

The second issue often encountered is the lack of a suitable computing infrastructure to perform the computationally intensive parts of the analysis. Sequence alignment in particular is very demanding in that regard. Even though many laboratories outsource the sequencing and alignment process, the possibility to realign the raw sequencing data is often required. To perform the described analysis steps and more, GensearchNGS provides a complete software suite focusing on optimized algorithms and usage of distributed computing for optimal usage of the existing infrastructure. All the analysis steps make usage of multiprocessor systems to speed up the analysis, whereas only sequence alignment using the custom GensearchNGS alignment algorithm uses distributed computing. The entire functionality is provided through an intuitive user interface to lower the technical know-how needed to perform NGS data analysis. GensearchNGS provides its own way on how the analysis is approached by providing the user with a patient centric user interface and favoring an interactive analysis approach instead of a fully automated analysis pipeline. The user interface is focused on providing the functionality required to perform the previously described analysis, without overwhelming the user with rarely used features.

## 2. Materials and Methods

GensearchNGS has been developed as a multiplatform application, running on Windows, Linux, and OSX, using Java 6+. The application is a locally installed software and is not web based. In order to perform NGS analysis steps, GensearchNGS includes several existing external tools through plugins. Those tools are mainly used during the sequence alignment phase, giving the user the choice between different alignment algorithms which may have specific advantages related to the data to be analysed. The external alignment algorithms supported through plugins are BWA [7] and Bowtie 1 + 2 [8] as well as Stampy [29]. GensearchNGS can either use locally installed versions of those external tools or download and update them automatically. If the tools are installed by the user he can update them when desired; otherwise the same update mechanism used to update GensearchNGS itself is used to update the external tools. For sequencing data, GensearchNGS supports various input and output file formats. For raw sequencing data, the standard FASTQ format as well as FASTA, SFF, and FNA is supported. All of them are converted to the standard FASTQ file format during the data import phase. As FASTQ files require a quality score for every nucleotide in the sequenced reads, a default quality score is assumed for all the nucleotides if the source format, like FASTA, is missing this information. For aligned data, both SAM and BAM files are supported, whereas SAM files are converted to BAM during import. BAM and SAM file are accessed through the HTSJDK library. HTSJDK is an open source Java library, developed as part of the samtools project [12]. It provides a standardized API to access various NGS data file formats. The library can be downloaded at http://samtools.github.io/htsjdk/. Annotations can be provided through the BED, WIG, and GFF files and the VCF file format is used to import or export variants.

To benefit from the knowledge stored in various online databases, GensearchNGS has been connected to several of them to annotate the NGS data on various levels, from the genes and their transcripts down to the individual variants. For most of the annotation data, GensearchNGS accesses the Ensembl [24] web service through its biomart API, giving the application access to gene and variant information. Ensembl provides a unified way to access several other online databases, like dbSNP [16] or OMIM [30], through a single online service. In addition to Ensembl, GensearchNGS integrates other online services for additional data and features: Cafe Variome [31] provides a standardized way to share variants between various laboratories, UCSC [32] provides access to the human reference sequence, and HPO [18] provides information about phenotypes associated with genes. In situations with a direct conflict in the information between the different databases, where possible, the information with the most severe clinical impact is displayed. An example of this is contradictory SIFT [33] and PolyPhen 2 [34] scores for variants. By default the most severe will be shown to the user, with the possibility to manually get more information when requested. GensearchNGS also integrates the possibility to talk to other software installed by the user, such as Alamut [35], allowing the user to easily open a variant found by GensearchNGS inside a local Alamut installation.

## 3. Results

This chapter discusses how GensearchNGS implements the different analysis steps and how it tries to lower as much as possible the technical know-how and computing infrastructure required for NGS data analysis.

### 3.1. Software Suite

GensearchNGS provides the user with the possibility to create different projects to group the analysis of various patients based on their type or their relation. While many other NGS analysis tools provide the possibility to organize the data in projects, GensearchNGS puts the notion of patients and their associated metainformation in the center of the project management. Every project contains one or more patient and each can have several raw sequencing data associated. The raw sequencing data can in turn have several alignments associated, for example, using different aligners or alignment configurations. Project specific settings such as specific regions of interests to be used during the analysis can also be configured. Those regions can be described by BED files to, for example, limit the analysis to regions relevant to the type of analysis performed in the current project. Those BED files can either be provided by the user or automatically generated based on a list of gene names. Projects also associate the specific reference sequence to be used during the analysis. The user can automatically download them from UCSC, which provides reference sequences of the most common human reference assemblies, hg18, hg19, and hg38. The usage of custom references sequences is also supported. The patient centric database integrated into GensearchNGS allows the user to associate variants with patients, a feature very important in diagnostics. The variants associated with patients are shared between projects, allowing the user to identify variants found in different projects on previously analysed patients.

**Figure 2:**
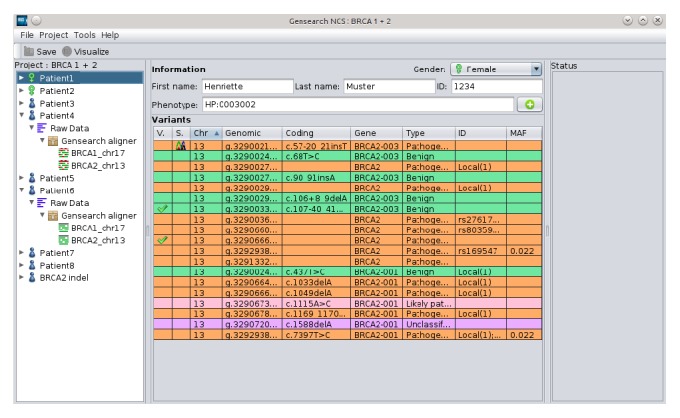
Main interface of GensearchNGS, showing a patient with its validated variants.

To perform the NGS data analysis, GensearchNGS uses the genes described in Ensembl as the gene model. In a process transparent to the user, the gene model corresponding to the reference sequence defined for the analysis is downloaded automatically from Ensembl. Additional metainformation is attached to the genes, such as the associated phenotypes as described by HPO [18] or OMIM [30].  Figure 2 shows the main interface of GensearchNGS, with the patient list on the left and the information about the currently selected patient in the center. The central area of the image shows the information about the currently selected patient. This includes his name, internal ID, associated phenotypes, and all variants which have manually been confirmed by the user. When validating variants the user can classify them manually into different categories. The categories are based on the ACMG variant classification guidelines [36], with the user requested categories* Artefact* and* False reference* added. The saved variants are used for any future variant detection in new patients, immediately telling the user if a variant has already been found in a different project. The addition of the* Artefact* and* False reference* categories allows the user to quickly filter out common sequencing errors in his particular sequencing setup. The hierarchical organization of the data is intended to guide the user through the analysis process while keeping it clear where the data comes from. An example of this is the sequence alignment. It is displayed as a child element of the raw sequencing data, making the relation between the raw data and the alignment immediately obvious.

GensearchNGS does not provide the user with a single button which goes from the raw sequencing data to the final report. Instead of fully automating the pipeline, GensearchNGS leads the user from one processing step to the next. Before each step the user is given the choice to change parameters or use the previously used (or standard) parameters. After the initial import of the raw sequencing data, the user no longer has to handle the data files. GensearchNGS performs the required data file handling and conversions between the processing steps.

While there is no fully automated way to go from the raw sequencing data to the final variant report, there is still a way to automate a big part of the analysis. When importing raw sequencing data, the user has the possibility to perform sequence alignment and variant calling automatically. Those two additional analysis steps are performed using either previously used configuration parameters or the default ones. Only the final variant validation has then to be performed by the user to create the final report. Further details of this process follow in the next chapter.

### 3.2. Data Analysis

**Figure 3:**
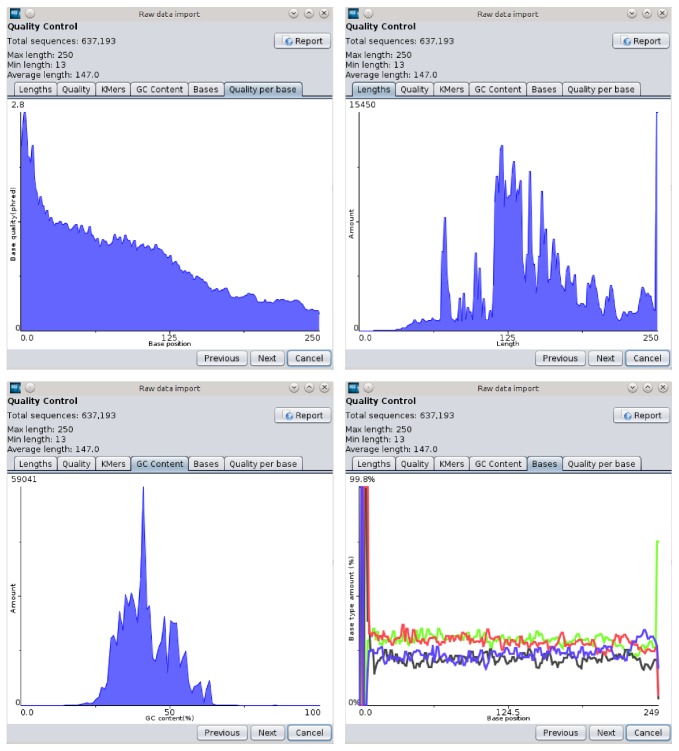
Overview of various statistics that the user can inspect to verify the data quality.

To perform the initial quality control of the raw sequencing data, GensearchNGS integrates its own tools allowing the user to import the raw data available in different file formats. The compatible data formats are described in Section 2. The various formats are converted in the standard FASTQ format during the data import step. For the quality control of the raw sequencing data, the user is presented with various statistics as seen in Figure 3.

The statistics allow the user to judge the quality of the sequencing data. This includes statistics such as the length, average quality, and GC content distribution of the sequenced reads, as well as the distribution of the sequenced bases and their quality for every read position and detection of overrepresented K-mers at the start and the end of the sequenced reads.

This raw data import process replaces existing tools like FastQC to check the quality, FASTX to filter the data, and the various utilities that transform different sequencing file formats into the standard FASTQ file format. Transparently for the user, the custom implementations of those tools, which produce the same results, have been integrated into a single processing step. This includes the possibility to remove adapter sequences and split the raw sequencing data into multiple files if they were produced through multiplexed sequencing. Based on the reported quality information, the user can choose to filter the raw data following several filters, such as maximum and minimum length of the sequences and their minimal average quality. He can also trim the 5′ and 3′ parts of all reads or trim their excess length if they are longer as a user defined length. The trimming is done in addition to the adapter and primer removal. The possibility to split the raw sequencing data based on barcodes in the read, allowing the import of raw data from multiple samples that were sequenced at the same time, is also integrated in the import process.

To align the sequenced data against a reference assembly, GensearchNGS provides the user with many options. Several existing alignment algorithms are integrated into the application through plugins, allowing their usage without the need of advanced technical know-how. The currently integrated alignment algorithms are BWA [7], Bowtie 1 + 2 [8], and Stampy [29].

Most of those alignment algorithms are developed in a way that do not allow them to be used on multiple platforms. In fact, most of them only run on Linux, which is problematic for a multiplatform application like GensearchNGS. For this reason, a custom alignment algorithm written in Java has been integrated directly into GensearchNGS. This allows the user to perform the full NGS data analysis, even on operating systems, like Windows, not supported by most bioinformatics tools.

The aligner supports paired end reads and gapped alignment and has been optimized to use less than 5 GB of RAM to perform a full genome alignment. This makes it suitable for most of today's workspace desktops. It is fully multithreaded to support all cores of the machine and is able to offload calculations, as described in Section 3.4, to multiple computers. The aligner uses a hash based index, a concept made popular through the BLAST aligner [9], with a custom heuristic based candidate alignment positions selection [37]. The final alignment is being done using the Gotoh [38] algorithm, a dynamic programming algorithm used for gapped alignment.

The custom alignment algorithm being implemented in Java does not achieve quite the same performance as natively running aligners like BWA. For WGS datasets it is generally 50% slower. The advantage of the GensearchNGS aligner over external aligners is that it is available on all supported platforms, allowing the user to perform the complete NGS data analysis on all platforms. But when running on a platform which supports the external aligners and speed is a deciding factor, the users are encouraged to use the aligners available as plugins.

The quality of resulting alignment can be verified through various metrics by the user. He can easily verify how many sequences have been discarded during the alignment process and how many sequences have been aligned to the different chromosomes in the reference sequence. Advanced statistics, similar to the ones created by bedtools 2, can also be inspected, giving detailed coverage information of the previously defined regions of interest. The collected quality control information can be exported as a report for archiving purposes using one of various formats such as PDF, HTML, or PNG.

While initially GensearchNGS integrated Varscan 2 [14] as its variant caller, a replacement was created to reduce the external dependencies and to improve the performance of variant calling. A variant scanner based on the model of Varscan 2 has been implemented and is in the process of being published as a separate tool called GNATY, available for free for noncommercial usage (http://gnaty.phenosystems.com/). Our custom variant caller shows important performance advantages over the existing Varscan 2 implementation of the same variant calling model, while producing the same analysis results. Indeed, GensearchNGS shows speed improvement of up to 18 times for variant calling compared to Varscan 2, providing a similar range of features, such as filters that exclude sequencing artifacts.

To be able to further filter down the detected variants based on their relevancy to the sample's phenotype, several sources are used to annotate them with various information. This includes the prediction of the variant effect on the different transcripts it affects, a feature closely modeled after the Variant Effect Predictor (VEP) from Ensembl [17]. GensearchNGS implements its own version of the the VEP algorithm to be able to perform the effect prediction in a fraction of the time compared to the online VEP service. This is is mainly achieved by doing all calculations locally without having to transfer the data to a remote server. Predictions such as the addition or removal of a stop codon in an affected transcript, if a splice site is affected and many others, can be made with this algorithm. Additional information, such as if the variant is known in any public database, associated clinical significance, and SIFT [33] and PolyPhen 2 [34] predictions are retrieved through the Ensembl web service. Other services, like the Human Phenotype Ontology [18], provide the information about the phenotype associated with a specific gene, a crucial information in diagnostics.

**Figure 4:**
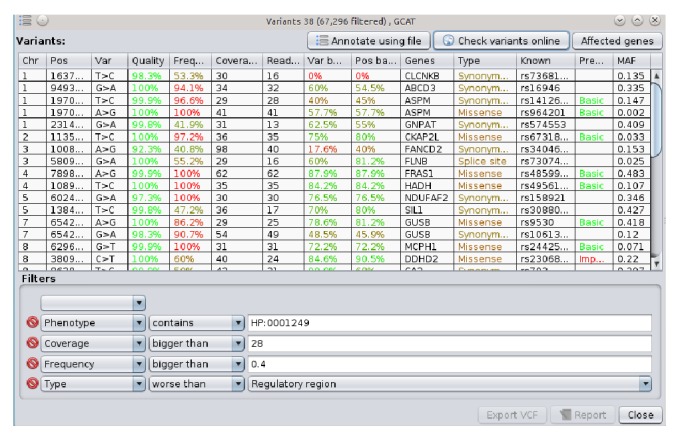
Integrated variants list with several interactive filters applied.

To filter the annotated variants, GensearchNGS takes an interactive approach which allows the user to modify different basic filters, such as minimum or maximum sample frequency, coverage, read balance, or quality. There are also advanced filters, such as the predicted consequence of the variant, as determined by the annotation process, or if a certain phenotype is associated with the affected genes. This includes the possibility to filter by population frequency of a variant. The filtering by population frequency does not yet take into account the ethnicity of the patient.  Figure 4 shows an example of this list, with several user selected interactive filters applied.

Additional features are available to perform a more in-depth analysis of variants, such as family analysis where the variants of multiple patients can be compared to find those only present in a certain group of patients. The usage of pedigree information is of growing importance in NGS data analysis, as it allows the user to filter sequencing artifacts and noncausative variants. This is a very important feature, especially when using WGS where the amount of variants can be very large. Thanks to the usage of standard file formats like VCF, external tools like exome walker [39] can be used to perform more advanced family analysis for features not covered in GensearchNGS. In contrast to many other NGS data analysis software packages, GensearchNGS does not focus on the automation of every aspect of the analysis. While certain parts can be automated, such as performing sequence alignment and variant calling on raw sequencing data, the philosophy of our software is to let the user to control the different analysis steps. This gives him enough flexibility to adapt to the data he is currently analysing without slowing him down, by having project specific analysis options which are preselected for every analysis step. This makes it possible to perform most types of analysis with the project specific default options through a simple press of a button.

### 3.3. Genome Browser

**Figure 5:**
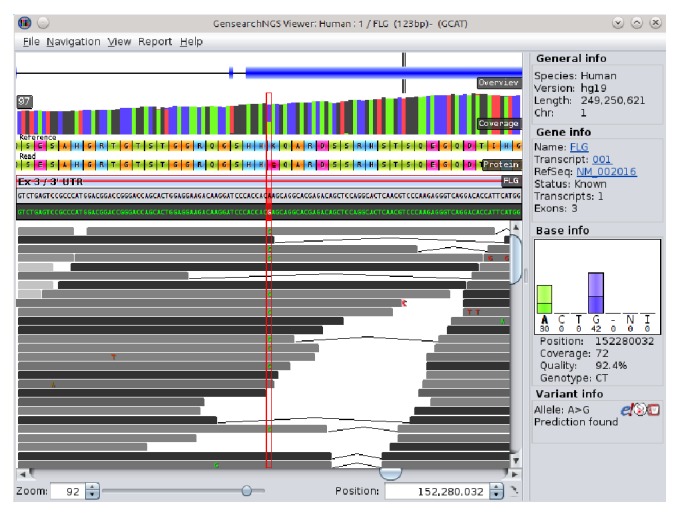
Example view of the genome browser, showing several available tracks.

A central tool of GensearchNGS is the integrated genome browser to visualize the aligned sequencing data. The genome browser, as shown in Figure 5, allows to interactively browse the sequence alignments. It is possible to display the aligned sequences and additional genomic data, such as the gene locations and the encoded proteins by different transcripts.

The genome browser allows the user to display several tracks, containing information which helps to analyse the sequencing data. The predefined tracks, which can be activated or deactivated, allow the user to display coverage information, GC content of the reference sequence, the quality of the sequenced reads, or custom annotation files in standard file formats such as BED, WIG, and GFF. The focus was put on optimizing the display of the data, allowing the user to visualize large datafiles without requiring a powerful computer. This is achieved by streaming the data on demand, only loading the parts of the data currently needed for the visualization. The information about which variants are present in the alignment is available to the user in the same form as the variant list in the main application. It can be seen in Figure 4, allowing the user to quickly jump to the variants of interest. The genome browser, like all of GensearchNGS, is heavily connected with external data sources (Ensembl, dbSNP, Entrez, OMIM, Blast, and HPO) providing the user with all relevant information when needed. The user can open external resources linked from many features in the visualized data, such as genes, transcripts, or variants, to retrieve additional information. The genome browser takes advantage of the integrated data sources, like the Ensembl gene model, to display and interactively update encoded proteins, based on the variants present in the visualized data. The genome browser is tightly integrated into GensearchNGS, allowing the user to easily visualize the alignment data belonging to features of the sample.

### 3.4. Distributed Computing

The lack of computing power to perform NGS data analysis is a very common issue in many laboratories. To address this problem, GensearchNGS does not only optimize the algorithms for speed and memory usage but also try to exploit the already existing computing infrastructure. One way to do this is to use distributed computing, a technique which allows the user to combine multiple computers to perform computing intensive tasks. Those computers can be in the same local network or at a remote location. One of the most demanding steps in NGS data analysis is sequence alignment. For this reason it is the first feature to take advantage of this technique in GensearchNGS, allowing the user to join multiple computers in the same network to perform sequence alignment. The approach used by GensearchNGS is to transparently, on request by the user, use all computers within the same network where GensearchNGS is installed. Additionally, the user has the possibility to start up GensearchNGS instances in a cloud. Currently, only the amazon cloud is supported. This process works regardless of the operating system used by the different installations of GensearchNGS. By using an automatic service discovery protocol, every installation of GensearchNGS located in the same network is discovered without the need for manual configuration. This allows the user to use multiple computers at the same time or to completely offload calculations to a different computer, making it possible to use computers not suited for full genome alignments as an analysis workstation. The distributed sequence aligner used in GensearchNGS currently only supports the GensearchNGS aligner algorithm and not external aligners such as BWA or Bowtie 2. It uses a stream based approach, where the client machine streams the data to be aligned to all computers involved in the alignment process. This includes the client machine which receives the aligned sequences back and combines them into the final alignment file. The process of distributing the alignments over multiple computers and the cloud is transparent to the user, but it is optional, because the usage of an external cloud is not compatible with the security guidelines of some laboratories. GensearchNGS is not the only NGS data analysis software using distributed sequence alignment. It is this ability to combine the computing resources of the local computer, multiple computers in the same network as well as the cloud, which makes its approach unique and very flexible.

## 4. Discussion

GensearchNGS addresses problems that smaller laboratories are facing in various ways. The issue of the technical complexity of the analysis is approached through an intuitive user interface. The user interface abstracts the complexity of the underlying NGS data analysis toolchain, which is a major usability improvement over the usage of individual tools. Nevertheless, it gives the user full control over the individual analysis steps, instead of focusing on the creation of automated analysis pipelines. It also provides an original approach by providing a patient centric user interface. This user friendly interface allows users unfamiliar with the underlying software tools to fine tune the analysis parameters, without overwhelming them with features or rarely used options. This increases the number of people able to perform NGS data analysis. This is particularly interesting for geneticists that might not have special bioinformatics training. The issue of increasing infrastructure requirements for NGS data analysis is being addressed twofold. First, more efficient algorithms are being developed, like the heavily optimized variant calling algorithm included in GensearchNGS. Secondly, the ability to distribute heavy calculations over several computers or even a cloud service has been developed. GensearchNGS has a unique approach to this problem by enabling the user to combine the various distribution methods to perform sequence alignment. It provides the flexibility needed to choose the best compromise between data privacy and alignment speed depending on the laboratory policies and needs. The resulting software enabled various laboratories to perform NGS data analysis and publish their results in various fields. The topics range from muscular dystrophies [40, 41] to hearing loss [42] and malignant hyperthermia [43] but also yet unpublished work about CNV analysis and the detection of somatic mutations in cancer screening.

## 5. Conclusion

In this paper we reviewed the workflow used to analyze NGS data in a diagnostics environment. The reviewed workflow includes the detection of SNPs and short indels from panel, WES or WGS DNAseq data. We also presented issues faced by smaller laboratories trying to setup a NGS data analysis pipeline. We then presented GensearchNGS, a software suite integrating various algorithms to perform the discussed workflow. GensearchNGS is a commercially available software, distributed by Phenosystems SA. A demo version of GensearchNGS is freely available at http://www.phenosystems.com/. The two main issues, the technical complexity of the analysis and the computing infrastructure requirements, have been addressed as follows.

To reduce the technical complexity of the analysis, the user is provided with an intuitive user interface which guides him through the process of the data analysis. He is provided with the required tools to go from raw sequencing data the the final visualization and report generation for the sequenced samples, without having to learn complex bioinformatics tools. The interface abstracts the technical complexity from the user while keeping enough flexibility to adapt to different analysis protocols. By lowering the technical complexity of NGS data analysis compared to existing individual analysis tools, GensearchNGS expands the potential user base of the software.

To address the issue of the computing infrastructure requirements, GensearchNGS focuses on the optimal usage of existing computing resources. This is primarily achieved through the development of analysis algorithms that run on a minimal amount of resources while producing the same analysis results as standard tools. An example has been described with the variant calling algorithm which produces the same results as Varscan 2 but is 18 times faster. The second approach is the integration of distributed computing, allowing the distribution of computing intensive tasks like alignment to be speed up by the usage of multiple computers.

With a solid framework built for DNAseq analysis, GensearchNGS will expand into further types of analysis in the future. One example is the integration of RNAseq analysis, as the combination of OMICS data for a single sample is of increasing importance to understand rare diseases [44]. In fact, many parts of GensearchNGS, such as the genome browser, have already been adapted to support RNAseq data. The integration of multiple data sources will not only allow the user to integrate different types of OMICS data but also allow multiple views on the same sample. One example of this is the integration of Sanger sequencing to NGS data, a very useful feature for diagnostics where it is common practice to validate variants identified through NGS with Sanger sequencing.

Further optimizations of the algorithms used in the analysis will also be evaluated, including optimization for the underlying libraries. One example is HTSJDK, where several performance patches have been submitted and included, improving NGS data analysis performance not only for users of GensearchNGS, but also for the complete bioinformatics community.
